# The Clinicopathological and Prognostic Implications of FoxP3^+^ Regulatory T Cells in Patients with Colorectal Cancer: A Meta-Analysis

**DOI:** 10.3389/fphys.2017.00950

**Published:** 2017-11-21

**Authors:** Peipei Xu, Wei Fan, Zheng Zhang, June Wang, Ping Wang, Yirong Li, Mingxia Yu

**Affiliations:** ^1^Department of Clinical Laboratory, Zhongnan Hospital of Wuhan University, Wuhan, China; ^2^Department of Pathology, Zhongnan Hospital of Wuhan University, Wuhan, China

**Keywords:** colorectal cancer, FoxP3^+^ regulatory T lymphocytes, clinicopathological features, prognostic implications, meta-analysis

## Abstract

**Background and Objective:** Forkhead box P3 (FoxP3) is known as the specific marker for regulatory T lymphocytes (Tregs), which are responsible for self-tolerance and disturb the antitumor immunity. However, the prognostic implication of tumor-infiltrating FoxP3^+^ Tregs in patients with colorectal cancer (CRC) still remains controversial. The aim of this present study was to investigate the prognostic role of FoxP3^+^ Tregs in CRC through meta-analysis.

**Methods:** PubMed, Embase and Web of Science were searched for relevant articles up to December 12, 2016. Pooled hazard ratio (HR) and 95% confidence interval (CI) were calculated to explore the prognostic value of FoxP3^+^ Tregs in CRC. Odds ratio (OR) was calculated to investigate the correlation between FoxP3^+^ Tregs and pathological parameters.

**Results:** A total of 18 studies comprising 3,627 patients with CRC were enrolled in our meta-analysis. The combined HR for FoxP3^+^ Tregs on cancer-specific survival was 0.70 (95% CI = 0.62–0.80, *P* < 0.001). High FoxP3^+^ Tregs level was also associated with favorable prognosis on overall survival (HR = 0.76, 95% CI = 0.58–1.01, *P* = 0.058), with *P*-value very close to the statistical threshold. Yet, there was no correlation between FoxP3^+^ Tregs infiltration and disease-free survival (HR = 0.83, 95% CI = 0.63–1.09, *P* = 0.182). Moreover, FoxP3^+^ Tregs infiltration was significantly correlated with pT stage (OR = 0.50, 95% CI = 0.39–0.65, *P* < 0.001), tumor grade (OR = 0.77, 95% CI = 0.61–0.98, *P* = 0.032), lymphatic invasion (OR = 0.25, 95% CI = 0.07–0.89, *P* = 0.033) and vascular invasion (OR = 0.67, 95% CI = 0.52–0.86, *P* = 0.001).

**Conclusion:** The present meta-analysis suggests that high FoxP3^+^ Tregs infiltration is inclined to indicate favorable prognosis and is associated with the pathogenesis of CRC. Immunotherapy targeting Tregs in patients with CRC should be further investigated.

## Introduction

Colorectal cancer (CRC) is one of the most prevalent malignant tumors of the digestive track, with an estimated 134,490 new cases and 49,190 new deaths in the United States in 2016 (Siegel et al., 2016). During the past decades, despite advanced therapies including surgical resection, radiotherapy and neoadjuvant chemotherapy for patients have been obtained, CRC still remains the fourth most common cancer cause of death globally (Brenner et al., 2014). Recently, immunotherapy in patients with CRC has renewed scientific interest, such as checkpoint inhibition of cytotoxic T-lymphocyte associated protein 4 (CTLA-4), programmed cell death 1 (PD-1) and CD137 (Mazzolini et al., 2007; Correale et al., 2016). Gathering evidences indicate that tumor microenvironment (TME) could play an important role in tumor development and progression (Casey et al., 2015). For instance, it could hamper the function of natural killer (NK) cells and suppress monocyte-derived dendritic cell (MDCC) maturation (Michielsen et al., 2011; Coppola et al., 2015). Importantly, immunotherapy is confined due to the complexity of the TME and the interactions among tumor components (Michielsen et al., 2012; Becht et al., 2016). Thus, gaining a better understanding of the underlying mechanism of TME in CRC is eagerly awaited.

Regulatory T lymphocytes (Tregs), which typically express CD25 and account for 5–10% of CD4^+^ T cells, are an important component of the TME and play an essential role in maintaining immunological self-tolerance and disturbing the antitumor immunity (DiPaolo et al., 2005; Nomura and Sakaguchi, 2005). Forkhead box P3 (FoxP3), a transcription factor encoded by *FOXP3*, is known as the optimum marker for Tregs and conventionally thought to be indispensable for their development and function (Fontenot et al., 2003). The clinicopathological and prognostic value of FoxP3^+^ Tregs in patients with CRC has been a continuing topic of debate. Accumulating evidence demonstrated that, in the majority of solid tumors, a high density of tumor-infiltrating FoxP3^+^ Tregs predicted an impaired patient survival (Wolf et al., 2005; Hiraoka et al., 2006; Miller et al., 2006; Takenaka et al., 2013; O'callaghan et al., 2015). Paradoxically, not only no significant correlation between increased frequency of FoxP3^+^ Tregs and prognosis involving CRC patients was found in recent researches (Suzuki et al., 2010; Salama et al., 2012; Xu et al., 2013), but also several studies even reported that high FoxP3^+^ Tregs infiltration might be linked with a favorable prognosis in CRC (Reimers et al., 2014; Vlad et al., 2015; Argon et al., 2016; Chen et al., 2016).

In the light of the controversial statements mentioned above, we conducted this meta-analysis with an integrated large sample size to derive a more precise estimation of the prognostic value of FoxP3^+^ Tregs in patients with CRC. The relationship between tumor-infiltrating FoxP3^+^ Tregs and several clinicopathological features of CRC was also evaluated.

## Materials and methods

This systematic review and meta-analysis was conducted according to the guidelines of the Preferred Reporting Items for Systematic reviews and Meta-Analyses (PRISMA) statement (Moher et al., 2009).

### Search strategy

We searched PubMed, Embase and Web of Science for relevant studies published up to December 12, 2016, using the following terms: (colorectal cancer OR colorectal carcinoma OR colorectal tumor OR CRC) AND (Tregs OR regulatory T cells OR T regulatory cells OR FoxP3) AND (prognosis OR mortality OR survival). The articles were limited to human and English. Additionally, we searched the references of previously published reviews to identify additional relevant studies.

### Inclusion and exclusion criteria

In this meta-analysis, studies fulfilling the following criteria were eligible for inclusion: (1) studies were published as original articles; (2) patients did not receive any immunotherapy, chemotherapy or radiotherapy before surgery; (3) researches reported an association between FoxP3^+^ Tregs and cancer-specific survival (CSS), overall survival (OS), disease-free survival (DFS) or clinicopathological features; and (4) sufficient published data were provided to calculate hazard ratios (HRs) and 95% confidence intervals (CIs). When a single population was reported in multiple publications, only the report with the most complete data was included. Studies were excluded that considered potentially overlapping study samples, lacked sufficient data, or reviews, comments, case reports and conference abstracts. The analysis of FoxP3^+^ Tregs in tumor stroma was also an exclusion criterion.

### Data extraction

Two investigators independently selected articles and extracted data from eligible studies using a predefined form. From each study, the following information was extracted: first author's name, year of publication, country, the sample size, sample types, detection methods, markers of Tregs, cut-off definition, the clinicopathological features, the survival data (including CSS, DFS, and OS) and follow-up time. For survival data, if HR and corresponding 95% CI were not directly reported, they were estimated from Kaplan-Meier curves according to the method described by Tierney et al. (2007). In studies that reported a univariate and a multivariate analysis for the same comparison, we only used the latter. Any discrepancies were discussed and resolved by consensus.

### Statistical analysis

CSS was defined as the time from the initial diagnosis of CRC to death attributed to CRC. OS was defined as the time from diagnosis until death. DFS was defined as the interval between the initial primary diagnosis of CRC and the first relapse or death. HR and 95% CI were calculated to assess the association between FoxP3^+^ Tregs and survival. An observed HR > 1 implies a worse survival for the group with elevated FoxP3^+^ Tregs. Conversely, HR < 1 implies a favorable survival. Odds ratio (OR) was used for evaluating the association between FoxP3^+^ Tregs and clinicopathological features. Heterogeneity across studies was assessed using Cochran's Chi square-based *Q* test (Higgins and Thompson, 2002). If *I*^2^ ≥ 50% or *P* ≤ 0.05, the random effect model was applied to calculate pooled HRs or ORs. Otherwise, the fixed effect model was more appropriate. Furthermore, sensitivity analysis was performed to assess the stability of the pooled results. Publication bias was also investigated using Begg's test. All statistical analysis was performed by STATA 12.0 software (Stata Corporation, TX, USA) and *P* < 0.05 was considered statistically significant.

## Results

### Identification of eligible studies

**Figure 1:**
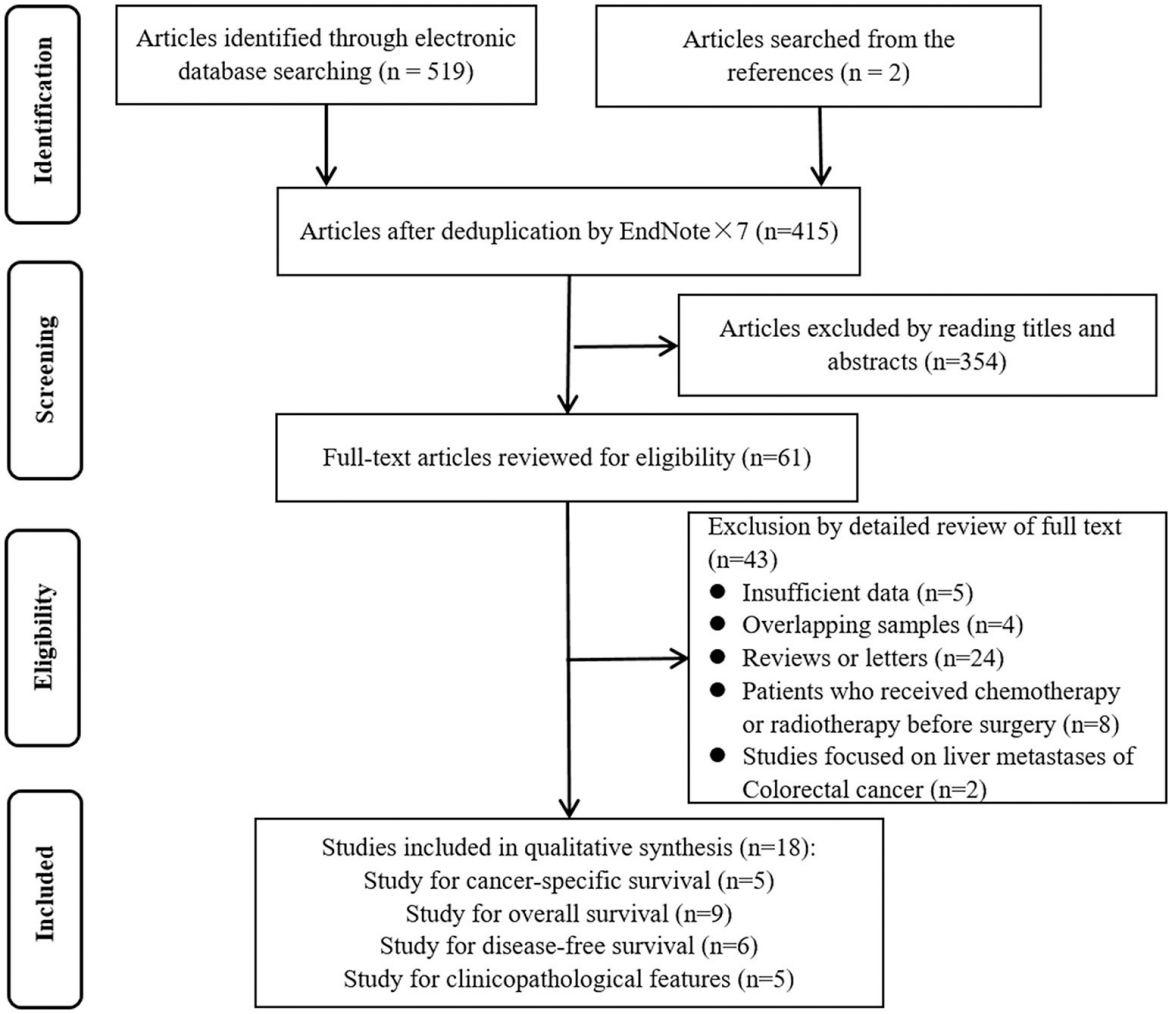
Flowchart of study selection.

A flowchart of study selection is presented in Figure 1. A total of 18 articles were included in our current meta-analysis (Frey et al., 2010; Lee et al., 2010; Suzuki et al., 2010; Sellitto et al., 2011; Salama et al., 2012; Kim et al., 2013; Xu et al., 2013; Chen and Chen, 2014; Ganapathi et al., 2014; Liu et al., 2014; Reimers et al., 2014; Zeestraten et al., 2014; Vlad et al., 2015; Argon et al., 2016; Chen et al., 2016; Markl et al., 2016; Saito et al., 2016; Zhu et al., 2016), including 5 on CSS (Frey et al., 2010; Sellitto et al., 2011; Salama et al., 2012; Vlad et al., 2015; Markl et al., 2016), 9 on OS (Lee et al., 2010; Suzuki et al., 2010; Kim et al., 2013; Xu et al., 2013; Chen and Chen, 2014; Ganapathi et al., 2014; Reimers et al., 2014; Argon et al., 2016; Chen et al., 2016), 6 on DFS (Lee et al., 2010; Suzuki et al., 2010; Reimers et al., 2014; Argon et al., 2016; Chen et al., 2016; Saito et al., 2016) and 8 on clinicopathological features (Frey et al., 2010; Lee et al., 2010; Kim et al., 2013; Liu et al., 2014; Reimers et al., 2014; Zeestraten et al., 2014; Markl et al., 2016; Zhu et al., 2016).

### Baseline characteristics of included studies

Table 1 summarizes the main characteristics of the eligible 18 studies. The analysis included 3,627 patients, ranging from 2010 to 2016. Among these studies, 8 were from Asia, 7 from Europe and 3 from Oceania. The most commonly used specimens were surgical tissues, while 2 studies detected Tregs in blood. The most commonly used test method for FoxP3^+^ Tregs was immunohistochemistry (IHC), besides, two studies used the quantitative real time polymerase chain reaction (qRT-PCR) and one used flow cytometry analysis (FCM). Tregs markers referred to FoxP3^+^ alone or combined with CD4^+^ and CD25^+^. The cut-off values of Tregs were not identical in different studies. In addition, the research by Frey et al. (2010) stratified the population into 3 groups, and we recorded all of these results as independent data sets.

**Table 1:** General characteristics of the studies included in this meta-analysis.

**Author**	**Year**	**Country**	**No. of patients**	**Specimens**	**Test methods**	**Markers**	**Cut- off value**	**Stage**	**Analysis index**	**Follow-up months**	**Analysis method**
Zhu	2016	China	48	Tissue	IHC	CD4^+^CD25^+^Foxp3^+^	>5%	I~IV	CP	NA	NA
Saito	2016	Japan	109	Tissue	qRT-PCR	FoxP3^+^	>median	II~IV	DFS	84	UA
Markl	2016	Germany	136	Tissue	IHC	FoxP3^+^	70 cells/mm^2^	I~II	CSS	55	UA
Chen	2016	China	300	TMA	IHC	FoxP3^+^	NA	II~IV	OS, DFS	62.9 ± 29.3	UA
Argon	2016	Turkey	186	Tissue	IHC	FoxP3^+^	NA	I~IV	OS, DFS	1-108	MA
Vlad	2015	Romania	42	TMA	IHC	FoxP3^+^	>162.25/mm^2^	II~III	CSS	NA	MA
Zeestraten	2014	Netherlands	285	TMA	IHC	FoxP3^+^	>median	I~IV	CP	NA	NA
Reimers	2014	Netherlands	495	TMA	IHC	FoxP3^+^	>median	I~IV	OS, DFS, CP	NA	MA
Liu	2014	China	63	Tissue	IHC	FoxP3^+^	NA	I~IV	CP	NA	NA
Ganapathi	2014	UK	60	Blood	qRT-PCR	FoxP3^+^	>median	I~IV	OS	48	MA
Chen	2014	China	102	Tissue	IHC	FoxP3^+^	>median	I~IV	OS	NA	UA
Xu	2013	China	90	TMA	IHC	FoxP3^+^	>median	I~IV	OS	65	MA
Kim	2013	Germany	65	Tissue	IHC	CD4^+^CD25^+^Foxp3^+^	>12%	I~IV	OS	>60	UA
Salama	2012	Australia	165	Tissue	IHC	FoxP3^+^	NA	II	CSS	72	UA
Sellitto	2011	Italy	47	Blood	FCM	CD4^+^CD25^+^Foxp3^+^	>4.48%	I~IV	CSS	26.6 ± 11	MA
Suzuki	2010	Japan	95	Tissue	IHC	FoxP3^+^	>14/10HPF	I~IV	OS, DFS	NA	UA
Lee	2010	Korea	87	Tissue	IHC	FoxP3^+^	>median	II	OS, DFS	125	MA
Frey	2010	Switzerland	507	TMA	IHC	FoxP3^+^	17cells/TMA punch	NA	CSS, CP	NA	MA
Frey	2010	Switzerland	541	TMA	IHC	FoxP3^+^	17cells/TMA punch	NA	CSS, CP	NA	MA
Frey	2010	Switzerland	204	TMA	IHC	FoxP3^+^	17cells/TMA punch	NA	CSS, CP	NA	MA

*No., number; TMA, tissue microarray; IHC, immunohistochemistry; qRT-PCR, quantitative real time polymerase chain reaction; FCM, flow cytometry analysis; FoxP3, Forkhead box P3; HPF, high-power field; CP, clinicopathological features; CSS, cancer-specific survival; OS, overall survival; DFS, disease-free survival; MA, multivariate analysis; UA, univariate analysis; NA, Not available*.

### The prognostic effect of FoxP3^+^ Tregs infiltration on survival

**Figure 2:**
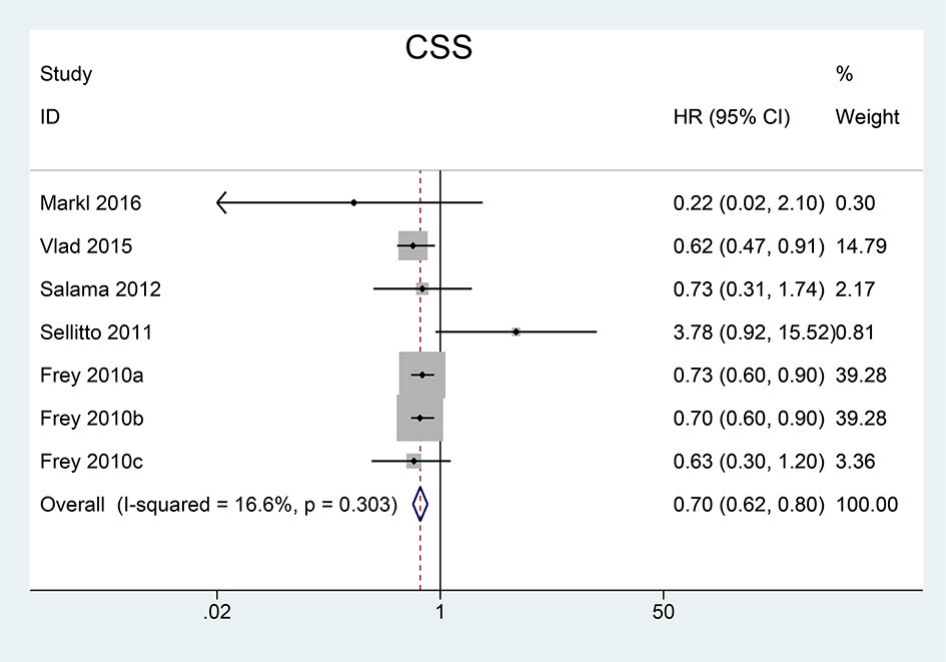
Forest plot of the HR for the association between FoxP3^+^ Tregs and CSS in patients with CRC. Frey et al. (2010) are for MMR-deficient group, MMR-proficient Test Group 1, and MMR-proficient Test Group 2, respectively. HR = 0.70, 95% CI = 0.62–0.80, *P* < 0.001, fixed model. FoxP3, Forkhead box P3; Tregs, regulatory T lymphocytes; CRC, colorectal cancer; CSS, cancer-specific survival; MMR, mismatch-repair; HR, hazard ratio; CI, confidence interval.

**Figure 3:**
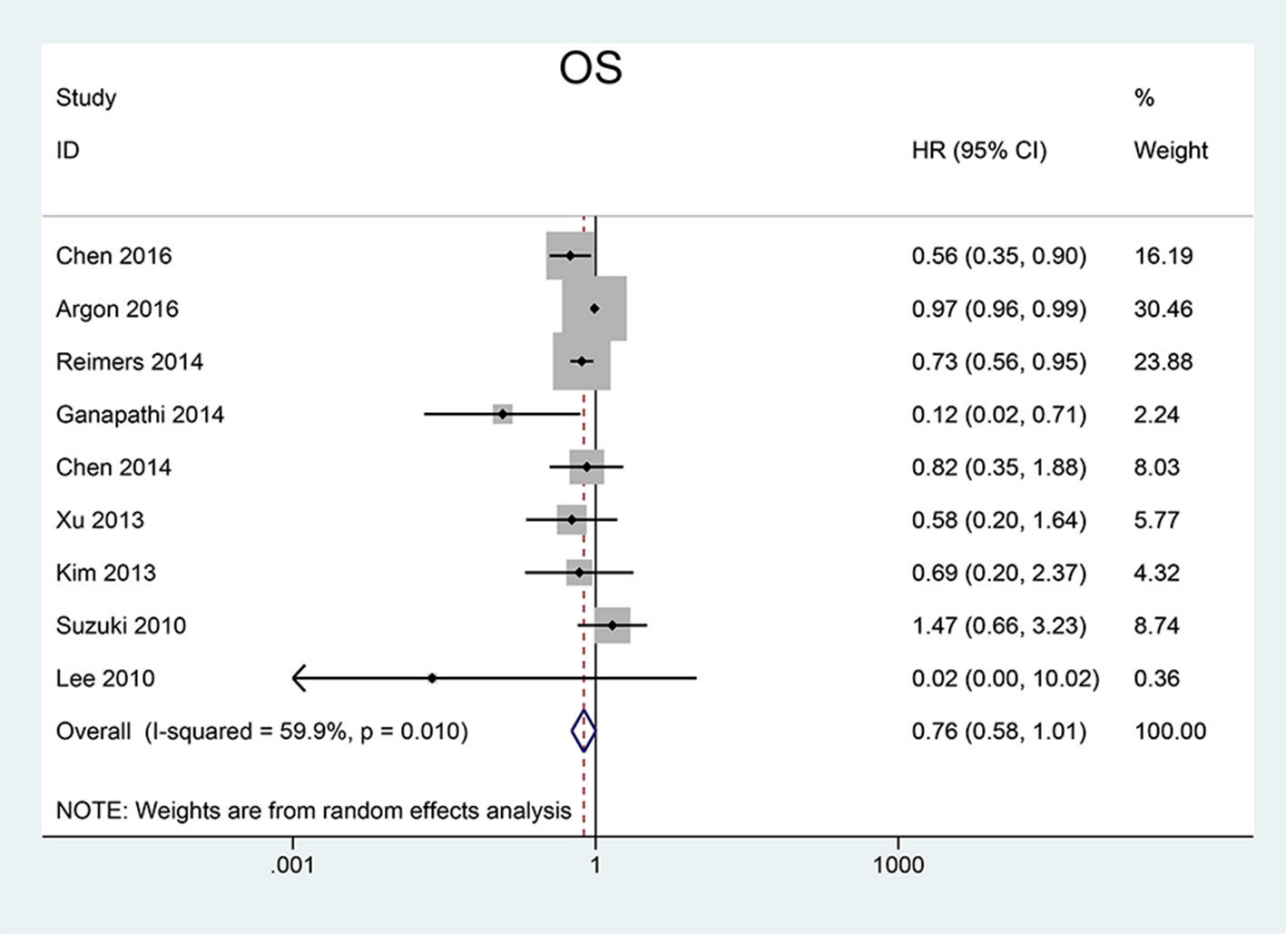
Forest plot of the HR for the association between FoxP3^+^ Tregs and OS in patients with CRC. HR = 0.76, 95% CI = 0.58–1.01, *P* = 0.058, random model. FoxP3, Forkhead box P3; Tregs, regulatory T lymphocytes; CRC, colorectal cancer; OS, overall survival; HR, hazard ratio; CI, confidence interval.

**Figure 4:**
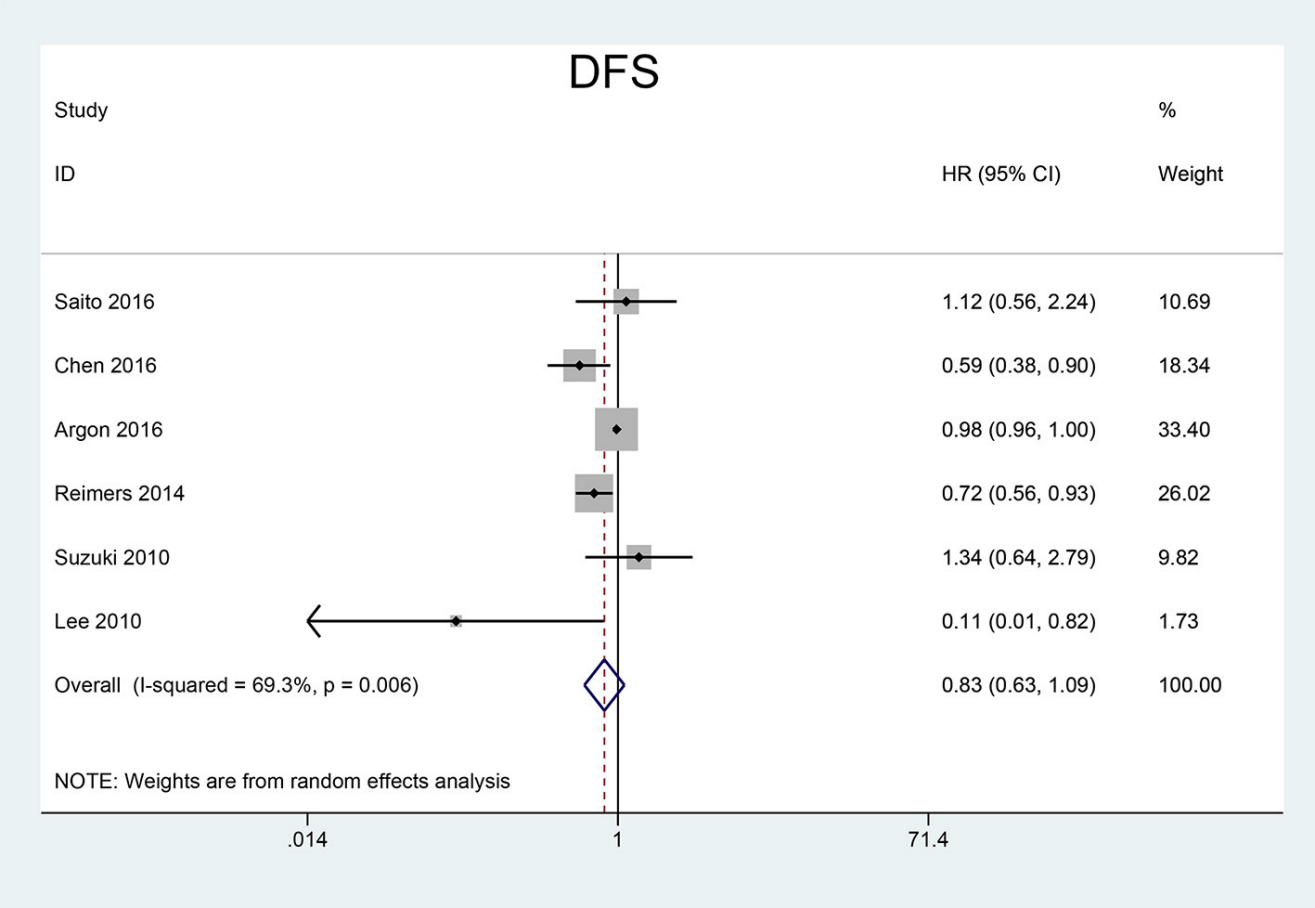
Forest plot of the HR for the association between FoxP3^+^ Tregs and DFS in patients with CRC. HR = 0.83, 95% CI = 0.63–1.09, *P* = 0.182, random model. FoxP3, Forkhead box P3; Tregs, regulatory T lymphocytes; CRC, colorectal cancer; DFS, disease-free survival; HR, hazard ratio; CI, confidence interval.

We pooled CSS, OS and DFS to assess the impact of FoxP3^+^ Tregs level on the prognosis of CRC. 5 studies reported a relationship between high Tregs infiltration and CSS, 9 studies published the data of OS while 6 studies on DFS. Our meta-analysis found that the CRC patients with high FoxP3^+^ Tregs infiltration showed superior CSS than those with low FoxP3^+^ Tregs infiltration (HR = 0.70, 95% CI = 0.62–0.80, *P* < 0.001, fixed model) (Figure 2). OS was slightly improved in patients with high FoxP3^+^ Treg infiltration (HR = 0.76, 95% CI = 0.58–1.01, *P* = 0.058, random model) (Figure 3), with the *P*-value very close to the statistical threshold. However, for DFS, the combined HR was 0.83 (95% CI = 0.63–1.09, *P* = 0.182, random model) (Figure 4), which indicates that FoxP3^+^ Tregs are not correlated with DFS in CRC.

### Correlation of FoxP3^+^ Tregs infiltration with clinicopathological feathers

To gain further insight into the value of FoxP3^+^ Tregs infiltration as an effective biomarker, we investigated the association between FoxP3^+^ Tregs level and certain clinicopathological parameters in patients with CRC. OR < 1 states that high level of FoxP3^+^ Tregs might be a favorable factor in the features.

As listed in Table 2, our results demonstrated that the high FoxP3^+^ Tregs infiltration were significantly associated with earlier pT stage (OR = 0.50, 95% CI = 0.39–0.65, *P* < 0.001), well or moderately differentiation (OR = 0.77, 95% CI = 0.61–0.98, *P* = 0.032), absence of lymphatic invasion (OR = 0.25, 95% CI = 0.07–0.89, *P* = 0.033) and absence of vascular invasion (OR = 0.67, 95% CI = 0.52–0.86, *P* = 0.001). There was no significant correlation in age (OR = 0.98, 95% CI = 0.54–1.78, *P* = 0.951), gender (OR = 0.95, 95% CI = 0.80–1.12, *P* = 0.533), tumor stage (OR = 0.96, 95% CI = 0.29–3.22, *P* = 0.947) and lymph node metastasis (LNM) (OR = 0.90, 95% CI = 0.51–1.57, *P* = 0.703).

**Table 2:** Association between high FoxP3^+^ Tregs infiltration and characteristics of patients with CRC.

**Factors**	**No. of studies**	**No. of patients**	**Pooled OR (95% CI)**	***P*-value**	**Heterogeneity**		**Statistical method**
					***I*^2^**	***P*-value**	
Age	3	442	0.98 (0.54, 1.78)	0.951	20.8%	0.283	Fixed
Gender	8	2,344	0.95 (0.80, 1.12)	0.533	35.5%	0.124	Fixed
pT stage	3	1,432	0.50 (0.39, 0.65)	<0.001	0%	0.787	Fixed
Stage	5	898	0.96 (0.29, 3.22)	0.947	90.4%	<0.001	Random
Grade	7	2,234	0.77 (0.61, 0.98)	0.032	34.6%	0.141	Fixed
LNM	4	1,387	0.90 (0.51, 1.57)	0.703	79.4%	<0.001	Random
Lymphatic invasion	2	199	0.25 (0.07, 0.89)	0.033	0%	0.994	Fixed
Vascular invasion	3	1,428	0.67 (0.52, 0.86)	0.001	0%	0.808	Fixed

*FoxP3, Forkhead box P3; Tregs, regulatory T lymphocytes; No., number; LNM, lymph node metastasis; OR, odds ratio; CI, confidence interval*.

### Sensitivity analysis and publication bias

**Figure 5:**
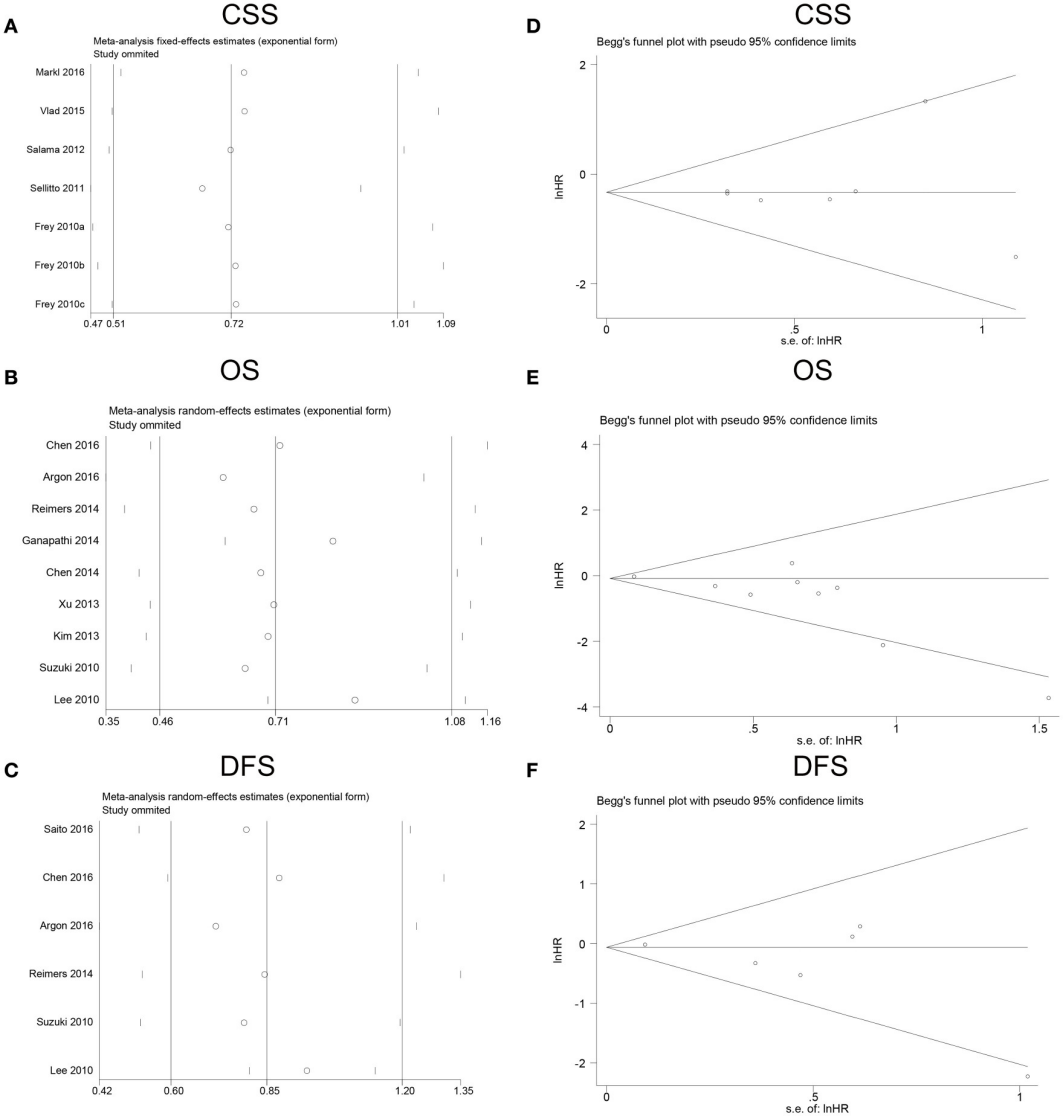
Sensitivity analyses and funnel plots for the publication bias of FoxP3^+^ Tregs on survival. **(A)** Sensitivity analysis for CSS; **(B)** sensitivity analysis for OS; **(C)** sensitivity analysis for DFS; **(D)** publication bias for CSS; **(E)** publication bias for OS; **(F)** publication bias for DFS. FoxP3, Forkhead box P3; Tregs, regulatory T lymphocytes; CSS, cancer-specific survival; OS, overall survival; DFS, disease-free survival.

All studies were sequentially omitted to explore that whether any individual study had a significant influence to the pooled HR. The recalculated HRs did not differ significantly from the overall value, demonstrating that our analyses were relatively stable and credible (Figures 5A–C). Begg's funnel plots did not indicate evidence of an obvious publication bias (Figures 5D–F).

## Discussion

The present meta-analysis demonstrates that high FoxP3^+^ Tregs levels are associated with a favorable impact on CSS in patients with CRC, whereas fail to assign prognostic robustness to OS and DFS. However, on OS, the *P*-value (0.058) was very close to the statistical threshold (0.05), suggesting that significance could have been reached with just a slightly larger patient population. The combined ORs indicate that FoxP3^+^ Tregs infiltration is significantly correlated with earlier pT stage, well or moderately differentiation, absence of lymphatic invasion and venous invasion. The results of sensitivity analysis indicate that the performance of FoxP3^+^ Tregs for prognosis in CRC patients is stable and reliable.

There were two relevant meta-analyses on this topic (Huang et al., 2014; Shang et al., 2015). In 2014, Huang et al. reported prognostic value of tumor-infiltrating FoxP3^+^ T Cells in gastrointestinal cancers. For CRC, the OS at 1, 3, and 5-year of high FoxP3^+^ T cells infiltration patients were higher than low FoxP3^+^ T cells infiltration patients (*P* < 0.001) and there were no differences in recurrences between high and low FoxP3^+^ T cells infiltration patients (*P* > 0.05) (Huang et al., 2014). In 2015, Shang et al. analyzed the prognostic value of FoxP3^+^ Tregs in different types of cancer, and they reported that high FoxP3^+^ Tregs infiltration had a significant positive effect on OS (OR = 0.71, 95% CI 0.62–0.82) and DFS (OR = 0.63, 95% CI 0.48–0.88) (Shang et al., 2015). The conclusion of our current meta-analysis was generally similar to previous meta-analyses on OS, but it was different from the meta-analysis performed by Shang et al on DFS (Shang et al., 2015).

Compared with previous meta-analyses above, our meta-analysis had several strengths to provide more convincible conclusions. Firstly, according to Cochrane handbook for meta-analysis, OR is not suitable for survival analysis with time-to-event data in consideration of censored data and time to study endpoint. So we used HR instead of OR to assess the survival analysis in the present meta-analysis. Secondly, the previous meta-analyses analyzed the overall survival combining CSS and OS, but according to their own definitions, we thought it was more rigorous to analyze them separately. Thirdly, two researches performed by Sinicrope et al. (2009) and Yoon et al. (2012) were included in the abovementioned meta-analyses. However, we found that the participants in these two studies had received preoperative chemotherapy. Considering that immunotherapy, chemotherapy and radiotherapy before surgery may modify the presence or the composition of T lymphocyte subsets through influencing immune reactions, we made an exclusion criterion to exclude these studies designedly. Fourthly, the samples of Suzuki et al. (2013) largely overlap with the study of Suzuki et al. (2010), we did not regard it was proper to take these two studies into dataset meanwhile. Fifthly, the relationship between tumor-infiltrating FoxP3^+^ Tregs and clinicopathological features was also evaluated in the present meta-analysis. We found that increased FoxP3^+^ Tregs infiltration was significantly associated with earlier pT stage, well or moderately differentiation, absence of lymphatic invasion and venous invasion. Though this finding cannot be simply interpreted as causal relationship between Tregs and better prognosis in CRC, it suggests that FoxP3^+^ Tregs are effective at delaying tumor invasion and progression. All in all, these five strengths significantly enhance persuasive power of the conclusions in the present study.

Our results suggest that higher FoxP3^+^ Tregs infiltration are inclined to indicate favorable prognosis on CRC. Nevertheless, to the best of our knowledge, high infiltration of FoxP3^+^ Tregs was a strong factor for unfavorable outcome in various solid tumors, such as breast cancer (Shou et al., 2016), non-small cell lung cancer (Zhao et al., 2016) and hepatocellular carcinoma (Zhao et al., 2014). Although this paradoxical phenomenon has existed all along, there are no conclusive explanations for it. Taking into account what is known in the literature, possibly the answer lies in the organ specific differences, the different TME in different tumors and functional activities of Tregs. Unlike other malignancies having a sterile microenvironment, abundant bacterial species were found to be enriched in CRC (Terzic et al., 2010). The bacteria often shift into colonic lumen and can infiltrate the tumor through the necrosis or ulceration of tumor surface (Soler et al., 1999). More seriously, many gastrointestinal bacteria can trigger the production of pro-inflammatory cytokines, followed by angiogenesis and tumor enhancing effects (Whiteside, 2012). However, the researches performed by Erdman et al. in mouse models of CRC demonstrated that adoptive transfer of Tregs was able to prevent bacteria-driven inflammation and carcinogenesis, when Tregs were previously exposed to enteric bacteria and could secrete IL-10 (Erdman et al., 2003a; Poutahidis et al., 2007). Meanwhile, the data suggested that IL-10 mediated suppression of host innate inflammatory response was pivotal in preventing or interrupting carcinogenesis (Erdman et al., 2003b). Thus, by directly suppressing inflammation and immune responses resulting from bacterial invasion, FoxP3^+^ Tregs could be in fact anti-tumorigenic in CRC. In addition, the relation between Tregs and pro-inflammatory immune cells like Th17 could also provide an explanation for the favorable effect of infiltrating FoxP3^+^ Tregs on CRC (Ladoire et al., 2011b). Th17 cells have pro-inflammatory effects through releasing cytokines. IL17, as an important cytokine from Th17, can induce angiogenesis through vascular endothelial growth factor (VEGF) (Murugaiyan and Saha, 2009). Thus, Th17 cells have mostly been thought to promote cancer growth. Because Tregs are known to inhibit activation and function of Th17 cells, in this way, we can demonstrate the relationship between Tregs and favorable prognosis. Furthermore, some authors claim that activated CD4^+^CD25^+^ Tregs may express granzyme and perforin and lead to the death of cancer cells via a perforin/granzyme dependent pathway (Grossman et al., 2004). To sum up, a high amount of Tregs is a good signal by hindering the development of CRC in diverse approaches.

Currently, downregulation or depletion the number of Tregs is a novel promising therapeutic strategy to enhance antitumor immune responses in some types of tumor. CTLA-4 and PD-1 pathways represent the two prevalent targets for therapeutic intervention (Voena and Chiarle, 2016). CTLA-4 is constitutively expressed in Tregs and upregulated upon initiation of T-cell receptor (TCR) stimulation, functioning as an immune checkpoint. Ipilimumab and tremelimumab, two inhibitors to CTLA-4, have shown a promising antitumor activity in patients with malignant melanoma. However, they showed poor results in the treatment of metastatic CRC (Buchbinder and Hodi, 2015). The preclinical findings and clinical responses associated with PD-1 and PD-ligand pathway blockade seem promising, making these targets highly sought for cancer immunotherapy. In fact, anti-PD-1/PD-L1 mAbs have received regulatory approval in multiple cancers, including melanoma, lung, kidney, bladder, and head and neck cancers, but they showed minimal activity in CRC (Shrimali et al., 2015). Thus, considering previous researches and our results, immunotherapy targeting Tregs in patients with CRC should be considered with caution and further investigated.

Our meta-analysis inevitably had some limitations. Firstly, the number of the included studies was relatively small and the included studies were only English researches, which might bring some publication bias. Secondly, the degree of statistical heterogeneity in our study was relatively large, which may be due to the different detecting techniques and cut-off values of FoxP3^+^ Tregs in the included studies. In addition, although it was known that FoxP3 was a specific marker for Tregs, some studies provided evidence that FoxP3 also expressed in tumor cells (Ladoire et al., 2011a; Tao et al., 2012; Kim et al., 2013). Thus, some potential inherent drawbacks might exist in experiments. Additionally, many studies had to be excluded because they did not report HRs and 95% CIs, but only with Kaplan-Meier curves. We attempted to reduce the missing data by estimating the outcome from Kaplan-Meier curves according to the method reported by Tierney et al. (2007). This may introduce some imprecision, but compared with excluding the studies, we felt this was a worthwhile risk. Therefore, the importance of a uniform report of study outcomes should be highlighted.

Despite these limitations mentioned above, our meta-analysis based on currently published articles has strengthened the evidence that higher FoxP3^+^ Tregs infiltration tends to be related to favorable prognosis on CRC. Considering our results, immunotherapy targeting Tregs in patients with CRC should be considered with caution and further studied.

## Author contributions

WF, MY, and YL conceived the study. PX and JW searched the databases and extracted the data. PX, PW, and ZZ analyzed the data. PX and ZZ wrote the draft of the paper. MY and YL reviewed and revised the manuscript. All the authors approved the final manuscript.

### Conflict of interest statement

The authors declare that the research was conducted in the absence of any commercial or financial relationships that could be construed as a potential conflict of interest.
